# Supplementary material for: Dysbiotic Gut Bacteria in Obesity: An Overview of the Metabolic Mechanisms and Therapeutic Perspectives of Next-Generation Probiotics
Source: Microorganisms. 2022 Feb 16;10(2):452. doi: 10.3390/microorganisms10020452 (PMC8877435; doi:10.3390/microorganisms10020452)
Supplement: Supplementary file 1 [file microorganisms-10-00452-s001.zip › microorganisms-1533505-Supplementary.pdf]

Table S1: Traditional probiotics efficacy to the care of obesity in animal and human clinical trials (not exhaustive).

				Pre-clinical evidence				Clinical evidence					
	Single Strain or Consortium	Composition	Weight gain	Food Intake	Fat accumulation	Inflammation	comorbidity	Body weight	Food Intake	Fat accumulation	Inflammation	comorbidity	References
Traditional probiotics	Single strain	*Lactobacillus gasseri BNR17*	↓**		↓		↓ insulin<br>↓ leptin	↓**		↓		↓ waist circumference	Kim et al. (2018)<br>Kang, J. H et al (2013)<br>SP Jung et al (2013)
	Single strain	*Bifidobacterium breve B-3*	↓		↓		↓insulin<br>↓ fasting glucose<br>↓ cholesterol			↓			S Kondo et al (2010)
	Single strain	*Bifidobacterium animalis subsp. Lactis CECT 8145*	↓	↓	↓		↓ plasma ghrelin levels<br>↑ insuline sensitivity	↓		↓		↓ waist circumference	NL Carreras et al (2018)<br>Pedret et al. (2018)<br>Cerdó, T. et al (2019)
	Single strain	*B. pseudocatenulatum CECT 7765*						↓					Cerdó, (2019).
	Single strain	*L. rhamnosus CGMCC1.3724*						↓					Sánchez et al. (2017) Cerdó, T. et al (2019)
	Single strain	*B. pseudocatenulatum CECT 7765*						↓					Sanchis-Chordá et al. (2018)<br>Cerdó, T. et al (2019)
	Consortium	*probiotic mixture including nine different strains of Lactobacillus and Bifidobacterium*						↓		↓			Szulinska et al. (2018)<br>Cerdó, T. et al (2019)
	Consortium	*L. acidophilus LA140, L. casei LC107, B. bifidum BBL730 and isoflavones*	↓		↓			↓					Cerdó, (2019).
	Consortium	*VSL#3 (Streptococcus thermophilus DSM24731, L. acidophilus DSM24735, L. delbrueckii subsp. Bulgaricus DSM24724, L. paracasei DSM24733, L. plantarum DSM24730, B. longum DSM24736, B. infantis DSM24737, and B. breve DSM2473)*						↓		↓		↓ insulin resistance<br>↑ GLP-1 level	Alisi et al. (2014)<br>Osterberg et al. (2015)<br>Cerdó, T. et al (2019)
	Consortium	*L. acidophilus ATCC B3208, L. rhamnosus DSMZ 21690, B. lactis DSMZ 32,296, and B. bifidum ATCC SD6576*						=				↓ insulin resistance<br>↑ GLP-1 level	Cerdó, (2019)
	Consortium	*L.curvatus HY7601 + L. plantarum KY1032*						↓		↓		↓ waist circumference	Jung et al. (2015)<br>Cerdó, T. et al (2019)
	Consortium	*L. acidophilus LA-14, L. casei LC-11,Lactococcuslactis LL-23, B. bifidum BB-06, B. lactis BL-4*						=				↓ waist circumference	Gomes et al. (2017)<br>Cerdó, T. et al (2019)
	Consortium	*L.curvatus HY7601 + L. plantarum KY1032*						↓		↓			Cerdó, (2019).

** Trend.
